# In Vitro Assessment of Salivary Pellicle Disruption and Biofilm Removal on Titanium: Exploring the Role of Surface Hydrophobicity in Chemical Disinfection

**DOI:** 10.1002/cre2.70082

**Published:** 2025-05-08

**Authors:** Wenji Cai, Azam Fayezi Sisi, Mohamed‐Nur Abdallah, Ashwaq A. Al‐Hashedi, Juan Daniel Gamonal Sánchez, Enrique Bravo, Hasna H. Kunhipurayil, Rubens Albuquerque, Zahi Badran, Mariano Sanz, Faleh Tamimi

**Affiliations:** ^1^ Faculty of Dentistry McGill University Montreal Canada; ^2^ Faculty of Dentistry University of Detroit Merci Detroit Michigan USA; ^3^ ETEP Research Group, Faculty of Dentistry Complutense University Madrid Spain; ^4^ College of Dental Medicine, QU Health Qatar University Doha Qatar; ^5^ Faculty of Dentistry of Ribeirão Preto University of São Paulo São Paulo Brazil; ^6^ Periodontology Unit University of Sharija Sharija United Arab Emirates

**Keywords:** biofilms, decontamination, hydrophobicity, peri‐implantitis, salivary pellicle

## Abstract

**Objectives:**

Peri‐implantitis is mostly caused by a pathological biofilm that forms through complex processes, initiated by the formation of the salivary pellicle on implant surfaces. Understanding the nature of these pellicles and biofilm and how to remove them is important for preventing peri‐implant infections and improving the success of dental implants. This study explores the characteristics of the salivary pellicle on titanium surfaces and assesses the effectiveness of different decontamination agents in eliminating the salivary pellicle and related microbial contaminations.

**Materials and Methods:**

Titanium surfaces were contaminated with salivary pellicles and pathological biofilms. The nature of the salivary pellicle was characterized using X‐ray photoelectron spectroscopy (XPS), surface proteomics, contact angle measurements, and fluorescence microscopy. We tested six commonly used decontamination chemicals (chlorhexidine, essential oil‐based mouthwash, citric acid, phosphoric acid, saline, and phosphate buffer saline) as well as newly proposed treatments such as surfactants and solvents (acetone, acetic acid, and Tween 20) for their capability to eliminate salivary pellicles and pathogenic biofilms from titanium surfaces.

**Results:**

The hydrophobic nature of the salivary pellicle on titanium surfaces limits the efficacy of commonly used hydrophilic solutions in removing pellicles and bacteria. Organic solvents and surfactants, particularly acetic acid and Tween 20, demonstrated superior effectiveness in removing the pellicle and biofilm. Acetic acid was notably effective in restoring surface composition, reducing microbial levels, and removing multispecies biofilms.

**Conclusions:**

The use of surfactants and solvents could be a promising alternative for the treatment of biofilms on titanium surfaces. However, further studies are needed to explore their clinical applicability.

## 1 Introduction

Despite their high success rate, dental implants can be vulnerable to infections by pathological biofilms, with several cases progressing to chronic inflammation and peri‐implantitis (Diaz et al. 2022). The prevalence of peri‐implantitis is estimated to be 19.53% at the implant level and 12.53% at the patient level.

Biofilms are complex communities of bacteria and extracellular matrix that colonize surfaces. These biofilms form through a process that starts with the exposure of the dental implant to the oral environment. This results in spontaneous adhesion of salivary macromolecules and bacteria on the implant surface, triggering the formation of a salivary pellicle that can eventually evolve into a mature bacterial biofilm (Larsen and Fiehn 2017; Siqueira, Custodio, and McDonald 2012).

Prevention and treatment of peri‐implantitis rely on decontaminating the implant surface, which involves removing biofilms and the salivary pellicle (Alani and Bishop 2014). Various commonly used chemical solutions for implant surface decontamination have been used on an empirical basis (Ungvári et al. 2010; Meyle 2012). These include, among others, chlorhexidine (CHX), hydrogen peroxide (H_2_O_2_), citric acid (CA), 35% phosphoric acid (PA), essential oil‐based mouthwash, phosphate‐buffered saline (PBS), and saline (Larsen and Fiehn 2017; Renvert, Roos‐Jansåker, and Claffey 2008; Mouhyi et al. 1998; Charalampakis et al. 2015; Widodo et al. 2016). CHX, essential oil‐based mouthwash, and CA have a successful track record in treating gingivitis and periodontitis (Sanz 2015), whereas H_2_O_2_ is frequently used in endodontics (Redanz et al. 2018) and PA is used in restorative dentistry (Cardenas et al. 2018). CA is a commonly used root canal irrigating solution in endodontics due to its ability to effectively eradicate the inorganic components of the smear layer, making it more efficient than other acids such as phosphoric acid, polyacrylic acid, and lactic acid (Meryon, Tobias, and Jakeman 1987). Despite their potential for biofilm removal from dental surfaces, no conclusive evidence demonstrates that these solutions could successfully decontaminate and promote re‐osseointegration of dental implants.

Although CHX exerts bactericidal effects on implant surfaces, it cannot reduce the amount of biofilm on the implants (Chin et al. 2007). A meta‐analysis showed that its role in nonsurgical management of peri‐implantitis cannot be confirmed (Liu, Limiñana‐Cañal, and Yu 2020). Similarly, CA can significantly decrease the viable microbial load on the implant surface alone or in combination with H_2_O_2_ (Dennison et al. 1994; Mouhyi et al. 2000), making it a promising clinical application. Saline solutions can reduce the bacterial load and establish favorable surface conditions for peri‐implant bone healing (Widodo et al. 2016), and some animal studies suggest that debriding infected implants with saline alone can improve re‐osseointegration capacity (Subramani and Wismeijer 2012). Also, a randomized clinical trial on the management of compromised implants showed no significant difference between the use of saline and more aggressive methods such as 35% PA (Hentenaar et al. 2017). However, the mechanism by which saline solutions decontaminate titanium remains unclear. Indeed, the interaction between the above‐mentioned chemical solutions and the contaminated titanium surfaces is not fully understood (Ungvári et al. 2010; Abu‐Ta'a et al. 2008).

The salivary pellicle forms on the surface of teeth and dental materials within a few minutes of their exposure to the oral cavity. The salivary pellicle initiates bacterial colonization, and given enough time, it will thicken into a mature biofilm. The acquired salivary pellicle consists primarily of proteins, lipids, and other macro‐molecules, such as carbohydrates, which can serve as binding sites for bacterial colonization (M. Hannig and Joiner 2006; Teixeira et al. 2006). Hence, removal of the salivary pellicle could effectively decrease later binding of potential pathogens and dental biofilm formation (Cukkemane et al. 2014). Therefore, antifouling interventions against salivary pellicle formation and primary bacterial colonization are of great relevance in preventing peri‐implantitis. Conversely, interventions that cannot decontaminate the salivary pellicle are unlikely to remove much thicker and stickier biofilms.

The composition of natural dental surfaces, namely, enamel and dentin, is significantly different from that of metallic dental implants (Abdallah et al. 2018; Chawhuaveang et al. 2021). Consequently, the behavior of the pellicle and biofilm that forms on a titanium surface may differ from that on dental surfaces such as enamel and dentin (Fischer and Aparicio 2021). As a result, it is possible that the solutions commonly used for dental hygiene might not be suitable for metallic surface decontamination. This study hypothesizes that the hydrophobic nature of the salivary pellicle on titanium surfaces limits the effectiveness of commonly used aqueous decontamination solutions. This would imply that organic solvents and surfactants could be more effective in removing these contaminants. To test our hypothesis, the primary objective of our study was to evaluate the effect of salivary contamination on titanium surface hydrophobicity. The secondary objectives were to (1) characterize the physicochemical properties of saliva‐coated titanium surfaces; (2) evaluate the effectiveness of aqueous solutions and organic solvents in restoring titanium surface properties and reducing bacterial load from saliva‐coated titanium surfaces; and (3) assess the potential of novel organic agents for removing microbial biofilms from the titanium surfaces.

We characterized the physicochemical properties of titanium salivary pellicle and tested several solutions typically used in dental care, including essential oil‐based mouthwash (Sanz 2015), 0.2% CHX (Renvert, Roos‐Jansåker, and Claffey 2008), 50% citric acid (Caba‐Paulino et al. 2020), 0.9% saline solution (Charalampakis et al. 2015), PBS, or 35% PA (Cardenas et al. 2018). Additionally, we proposed alternative chemical solutions that may be more effective for titanium decontamination and assessed them using a model of a pathological biofilm.

## 2 Methods

The study design was approved by the Research Ethics Board Committee of McGill University (14‐464 GEN), and informed written consent was obtained from all the volunteers.

### 2.1 Titanium Samples

Titanium (ultracorrosion‐resistant pure titanium, Grade 2, McMaster‐Carr, Cleveland, OH) was obtained (rectangular bars [6.4, 12.7, and 305.0 mm]) and cut into smaller sections (12.7, 6.4, and 6.4 mm) using an abrasive cutter (Delta AbrasiMet, Buchler, ON). To ensure standardized flat surfaces, the titanium sections were mirror‐polished using a water‐cooled trimmer, silicon carbide papers (240‐to‐800 grit, Buehler, Lake Bluff, IL, USA), and polishing cloths (Text Met and Chemo Met I Polishing Cloth, Buehler, USA) with a colloidal silica polishing suspension (Master Med; Buehler, USA) (Alageel et al. 2015). All samples were cleaned in an ultrasonic bath (FS20 Ultrasonic, Fisher Scientific, Montreal, Canada) sequentially with acetone, ethanol, and distilled water for 15 min each, and then dried overnight in a vacuum oven.

### 2.2 Titanium Surface Contamination With Saliva

Saliva samples were collected from three healthy nonsmoker volunteers without xerostomia or recent usage of antibiotics. The saliva donors refrained from taking any medication 3 months before the study and did not have active carious lesions or overt periodontal disease. Unstimulated saliva was drooled into sterile test tubes more than 2 h after brushing, eating, or drinking. Immediately after collection, the saliva samples were centrifuged (10,000 *g* at 4°C for 10 min) to remove cellular debris and minimize saliva turbidity (Schipper, Silletti, and Vingerhoeds 2007). Each titanium disc was then incubated in the collected saliva (2 mL) at 37°C to expose it to the full range of salivary components, such as proteins and possible oral microbiota (Koban et al. 2013). The samples were ultra‐sonicated (FS20, 40 kHz at room temperature) to remove any nonadherent or weakly adherent salivary components from the titanium surfaces, leaving behind only the part of the pellicle that was strongly adsorbed to its surface.

### 2.3 Titanium Surface Contamination With a Multispecies Static Oral Biofilm

The following bacterial strains were utilized in this study: *Cont* These strains were cultured on blood agar plates (Blood Agar Oxoid No 2; Oxoid, Basingstoke, UK) supplemented with 5% (v/v) sterile horse blood (Oxoid), 5.0 mg/L hemin (Sigma, St. Louis, MO, USA), and 1.0 mg/L menadione (Merck, Darmstadt, Germany) under anaerobic conditions (10% H_2_, 10% CO_2_, and balance N_2_) at 37°C for 1–3 days.

Pure cultures of each bacterium were collected and inoculated in a protein‐rich medium that consisted of brain–heart infusion (BHI) (Becton, Dickinson and Company, Franklin Lakes, NJ, USA) supplemented with 2.5 g/L mucin (Oxoid), 1.0 g/L yeast extract (Oxoid), 0.1 g/L cysteine (Sigma), and 2.0 g/L sodium bicarbonate (Merck) under anaerobic conditions for 24 h. Following incubation, bacterial growth was assessed by spectrophotometry (OD_550_ nm) until a bacterial mixture containing 10^3^ CFU/mL of *Streptococcus oralis*, 10^5^ CFU/mL of *Actinomyces naeslundii* and *Veillonella parvula*, and 10^6^ CFU/mL of *Fusobacterium nucleatum*, *Porphyromonas gingivalis*, and *Aggregatibacter actinomycetemcomitans* was obtained.

Clean mirror‐polished titanium discs were placed in the wells of a 12‐well tissue culture plate. Each well was inoculated with 1.5 mL of the above‐mentioned bacterial mixture. Wells containing the culture medium only were used as a check for sterility. All samples in the plates were incubated in anaerobic conditions at 37°C for 72 h. Once the 72 h biofilms were formed, all the titanium samples were rinsed in 2 mL of sterile PBS to remove nonadherent bacteria (Bravo et al. 2023).

### 2.4 Decontamination of Titanium Surfaces

Saliva‐coated titanium samples were decontaminated by ultra‐sonication in different chemical solutions for 15 min. The chemical solutions included six commonly used hydrophilic solutions (essential oil‐based mouthwash as Listerine, 0.2% CHX, 50% CA, 0.9% saline solution, PBS, or 35% PA), as well as three newly proposed treatments: two organic solvents (acetone, acetic acid) and one surfactant (Tween 20). Clean titanium discs without any saliva exposure served as controls.

Based on the results from salivary pellicle decontamination experiments, the two best‐performing solutions were selected to test their ability to clean titanium surfaces contaminated with pathological biofilms: CA and acetic acid. Titanium samples with multispecies oral biofilm were subjected to the following treatments (*N* = 3, repeated twice): (i) CA for 15 min, (ii) acetic acid for 15 min, or (iii) CA for 7.5 min, followed by acetic acid for 7.5 min. Clean titanium discs without any biofilm coating served as controls. After treatment, a subset of samples was assessed using the live/dead analysis, whereas another subset was vacuum‐dried and analyzed by X‐ray photoelectron spectrometry (XPS). To exclude the effect of chemical solutions or organic agents on the titanium surface, clean titanium discs were also directly exposed to the aforementioned test solutions for 15 min before analysis by XPS (Figure S1).

### 2.5 Contact Angle Measurements

The hydrophobicity of the titanium samples was assessed by static contact angle measurements recorded using the sessile drop method operated at room temperature, using a contact angle meter (OAC 15, Data Physics, Germany). The values were reported as averages of at least five drops (1 μL) of distilled water per sample. The measurements were captured using video‐based software (SCA 20, Dataphysics Instruments, Germany).

### 2.6 X‐Ray Photoelectron Spectroscopy

XPS is an extremely sensitive technique used to analyze the surface composition of the outermost atomic layer of a material. The surface elements of different titanium samples were characterized by a monochromatic XPS (K alpha, Thermo Fischer Scientific Inc., East Grinstead, UK) equipped with an Al Kα X‐Ray radiation source (1486.6 eV, 0.834 nm) and an ultrahigh vacuum chamber (10^−9^ torr) (Abdallah et al. 2016). Samples were vacuum‐dried overnight before XPS analysis. Survey scans were obtained with a pass energy of 100 eV at a step of 2.0 eV. High‐resolution scans of carbon (C1s) were collected with a pass energy of 50 eV at a step of 0.1 eV. The binding energy (BE) scale for the samples was calibrated by setting the value of the carbon bonded to hydrogen or carbon (C─ (C, H)) as a reference at 284.8 eV (Abdallah et al. 2014). Data analysis and peak fitting were performed using Advantage surface analysis software (5.41v, Thermo Fischer Scientific Inc., East Grinstead, UK).

### 2.7 Microbicinchoninic Acid (Micro‐BCA) Assay

BCA was used to compare the concentration of surface proteins that remained in different testing groups. In brief, we collected the proteins attached to each titanium sample in a well containing 1 mL of a solution containing 80% acetonitrile, 19.9% water, and 0.1% trifluoroacetic acid. This procedure was repeated three times to release all the adsorbed proteins from the surface (Siqueira et al. 2012). The eluted solution from each group was combined and concentrated by a rotary evaporator. The total protein concentration was determined using the Micro‐BCA assay (Thermo Fisher Scientific, USA). To determine the impact of ultrasonic bath only on the protein removal, saliva‐coated titanium before or after an ultrasonic bath in distilled water for 15 min was also compared by the BCA test.

### 2.8 Bacterial Live/Dead Assay for Contaminated Samples

Live/Dead assay and fluorescence microscopy were utilized to determine the bacterial load on the contaminated titanium sample and evaluate the effectiveness of the various cleaning solutions. The live/dead stain (BacLight Bacterial Viability Kit, Molecular Probes, Carlsbad, USA) was prepared by diluting 1 μL of SYTO 9 (excitation/emission (*λ*) = 485/498 nm) and 1 μL of propidium iodide (excitation/emission (*λ*) = 535/617 nm) in 1 mL of distilled water. The samples from each group were placed in a 12‐well plate, and 500 μL of the staining mixture was added to each well. They were incubated in the dark at room temperature for 15 min and then stored in a dark space at 4°C until further processing.

The saliva‐contaminated samples were examined using an upright fluorescence microscope (Carl Zeiss Microscopy GmbH, Gottingen, Germany) equipped with a digital camera (AxioCam MRm Rev. 3, Carl Zeiss Microscopy, Gottingen, Germany) and an image processing software (ZEN; Carl Zeiss Microscopy GmbH, Gottingen, Germany). A laser scanning confocal microscope (Leica SP9, Mannheim, Germany) was used to analyze the biofilm‐contaminated samples.

For each sample, three random sites were captured by a ×10 objective. The median of green/red fluorescence (live/dead cells) per microscopic field area of 0.15 mm^2^ was registered using cell profiler image analysis software (Broad Institute of MIT and Harvard, Massachusetts, USA).

**Figure 1:**
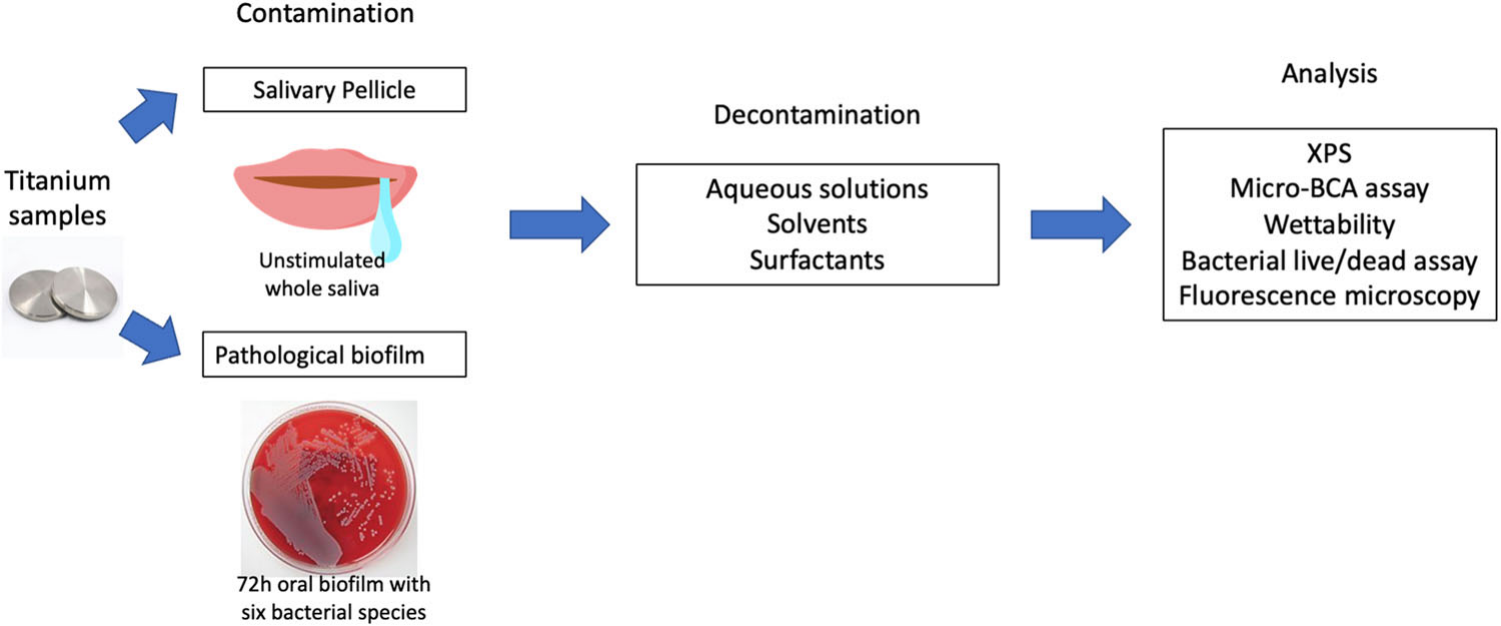
Workflow of the study design for assessing salivary pellicle and pathological biofilm decontamination on titanium surfaces. Titanium samples were contaminated with either salivary pellicle or pathological biofilm, followed by different decontamination methods and corresponding analysis.

Workflow of the study design for assessing salivary pellicle and pathological biofilm decontamination on titanium surfaces is illustrated in Figure 1.

### 2.9 Statistical Analysis

Statistical analysis was performed using Origin 9.0 (Origin Lab, Northampton, MA) and SPSS software (IBM SPSS Statistics 20, IBM Corporation, Somers, NY, US). The sample size (*n* = 6–9 per group) was determined based on practical experimental considerations and established norms in in vitro biomaterial research (Serdar 2021). The normality of data distribution was assessed using the Shapiro–Wilk test, which is well suited for small to moderate sample sizes due to its sensitivity and robustness. Based on the normality results, either one‐way ANOVA or the non‐parametric Kruskal–Wallis test was used to determine statistically significant differences between groups, with a significance level set at *p* < 0.05.

## 3 Results

### 3.1 Saliva‐Contaminated Titanium

**Figure 2:**
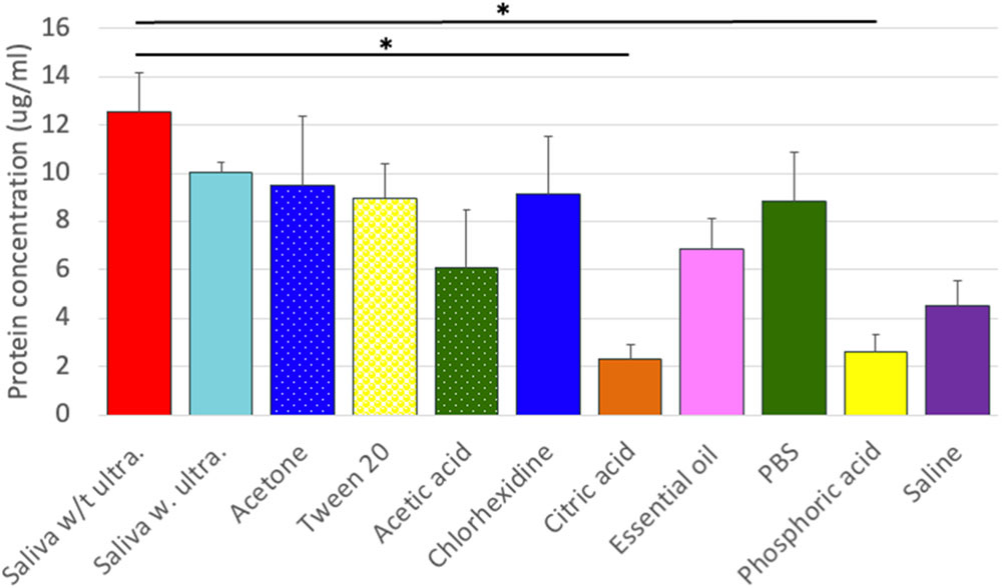
BCA assay demonstrated protein concentration of clean Ti samples after exposure to different solutions (*n* = 3 samples size per group) (*p* < 0.05).

Fluorescence microscopy, BCA, and XPS analyses demonstrated that titanium exposure to saliva resulted in the formation of a salivary pellicle rich in bacteria, proteins, and saturated carbon compounds. The BCA test indicated that salivary proteins firmly adhere to the titanium surface, as there was no significant change in the amount of surface protein before or after an ultrasonic bath in distilled water (*p* = 0.991) (Figure 2).

**Figure 3:**
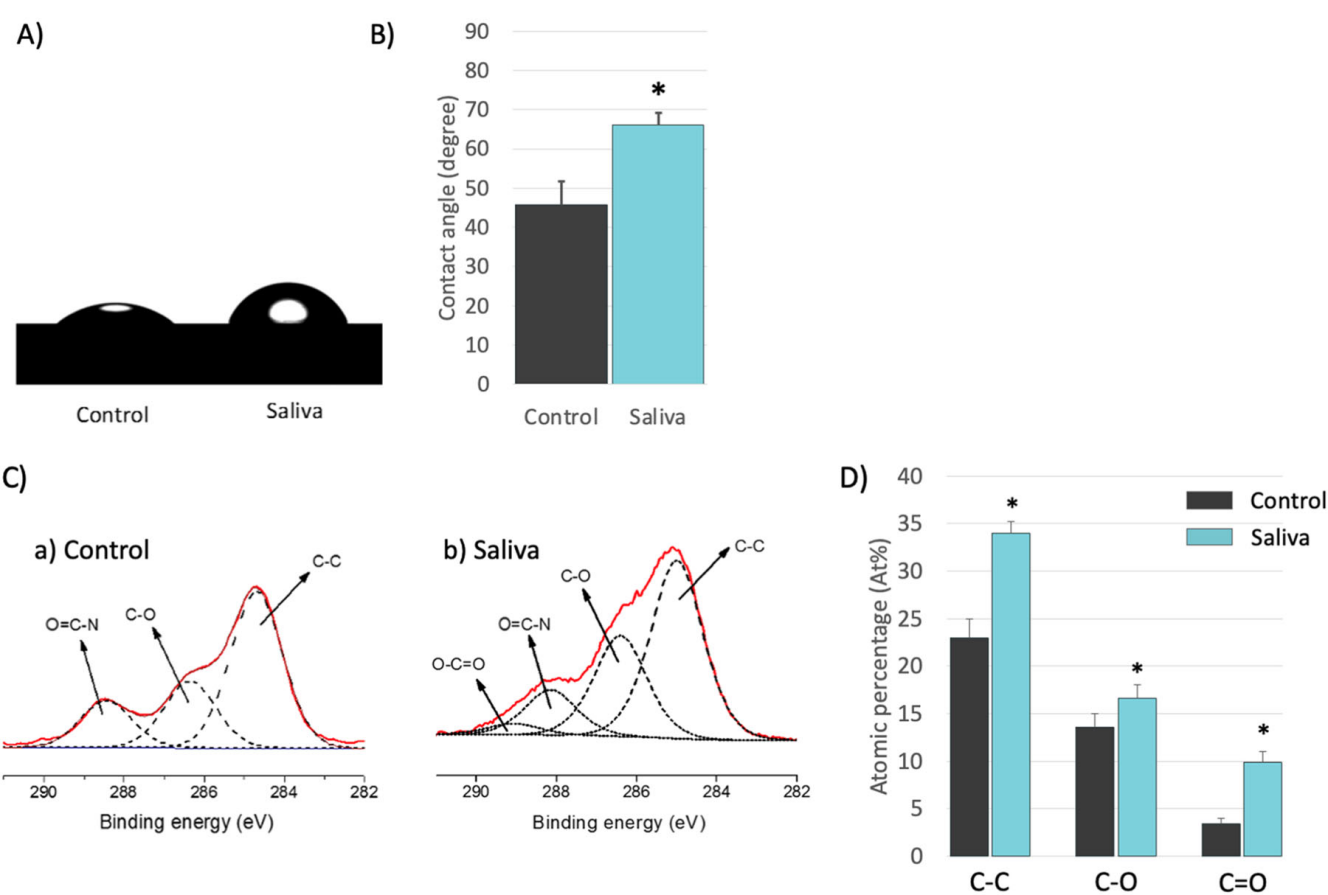
Effect of salivary contamination on the surface properties and composition of titanium. (A) Photographs of the contact angle experiment showing water droplets placed on a Ti surface before (control) and after saliva contamination. (B) Contact angle measurements showing a significant difference in the contact angle between the two groups (*p* < 0.05). (C) Peak fitting of the XPS high‐resolution C1s spectra on the Ti surface (a) before and (b) after saliva contamination. (D) Bar charts illustrating the atomic percentage of the different organic domains identified in the C1s spectra before and after saliva contamination on the Ti surface (*p* < 0.05).

The controlled polished titanium surface had a smaller contact angle (45.7° ± 14.8°) compared to the saliva‐coated samples (66.1° ± 3.1°) (Figure 3A,B).

**Figure 4:**
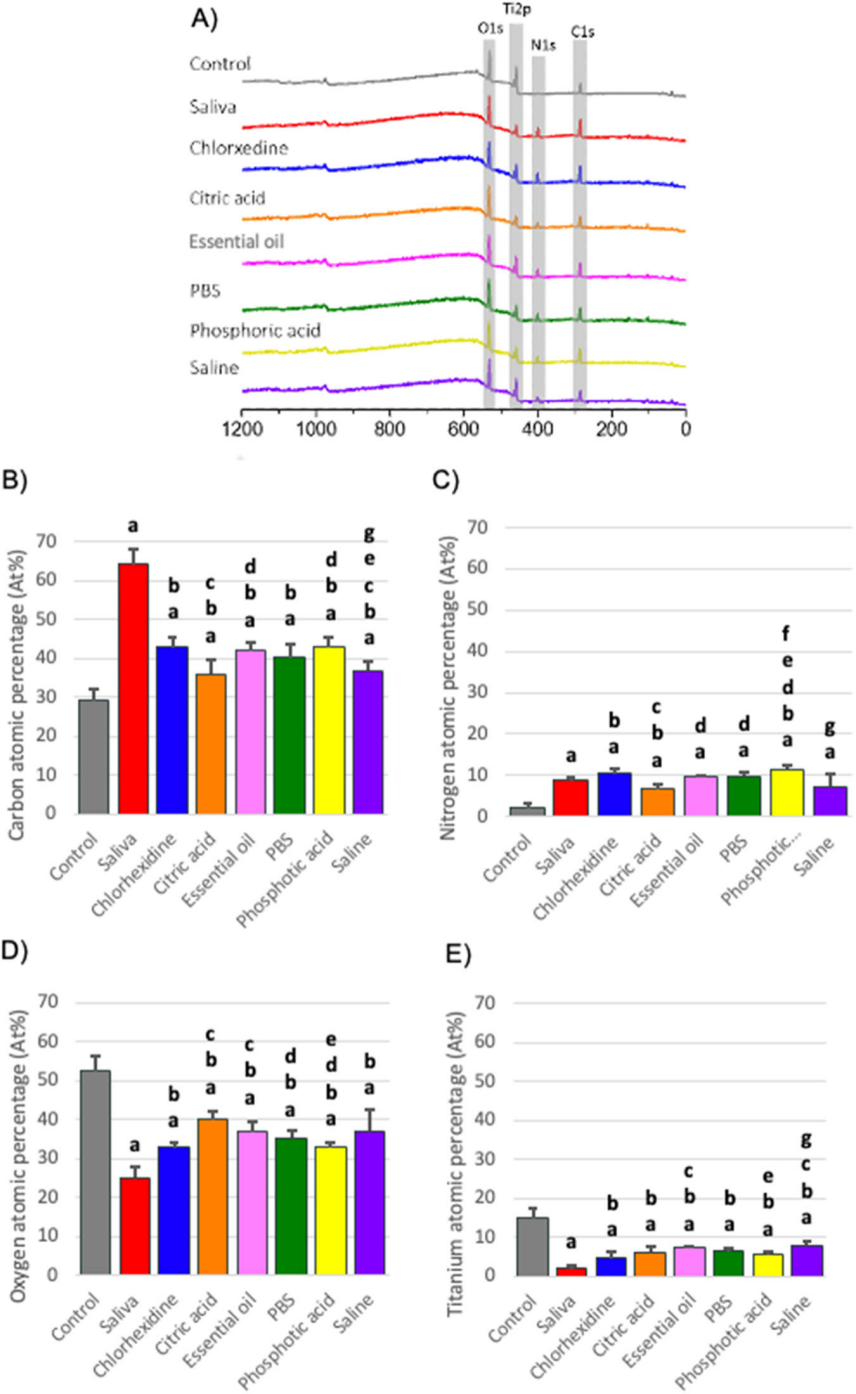
Effect of commonly used chemical treatments on the surface composition of titanium contaminated with salivary pellicle. (A) XPS survey spectra of the different surfaces: polished control Ti, and saliva‐contaminated Ti before and after treatment with cleaning agents (Listerine, chlorhexidine, citric acid, saline, PBS, and phosphoric acid). (B–E) Atomic percentage of carbon (C1s), nitrogen (N1s), oxygen (O1s), and titanium (Ti2p) on the different Ti surfaces; letters in the bar chart indicate significant differences from control (a), saliva (b), chlorhexidine (c), citric acid (d), essential oil‐based mouthwash (e), PBS (f), phosphoric acid (g), and saline (*p* < 0.05).

XPS analysis confirmed the presence of the salivary pellicle on the titanium samples by revealing significantly increased surface concentrations of carbon (C) and nitrogen (N), along with decreased concentrations of titanium (Ti) and oxygen (O) compared to the control, confirming an accumulation of organic components on the titanium surface (Figure 4A).

The C1s core‐level spectrum (red line) of the clean titanium (Control) or saliva‐coated titanium (Saliva) was curve‐fitted by distinct peaks at specific energy levels (Figure 3C,D). These peaks indicate the presence of aliphatic hydrocarbon (C─(C,H), BE at ~284.8 eV, carbon single‐bonded to oxygen or nitrogen (C─(O,N) at ~286.5 eV) probably from proteins, and carbon double‐bonded to oxygen (C═O at ~288.5 eV), which might be related to the ester bond (O─C═O) or the amide bond (O═C─N) (Beamson 1992).

### 3.2 Effect of Commonly Used Decontamination Aqueous Solutions on the Salivary Pellicle

**Figure 5:**
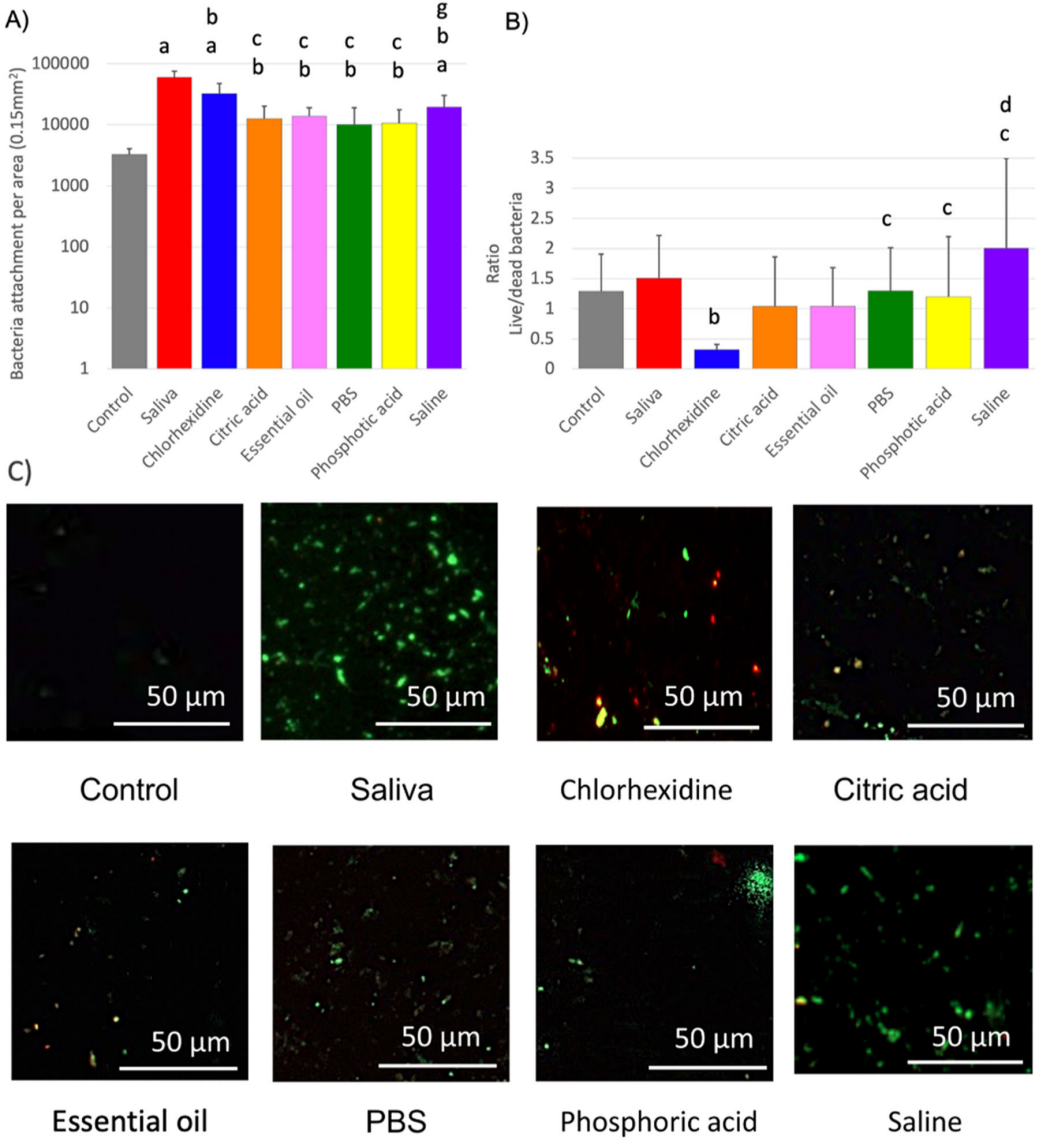
Effect of commonly used chemical treatments on the bacterial load of titanium surfaces contaminated with salivary pellicle: (A, B) Column bars show the results of fluorescence microscopy analysis to assess the microbial load on the surface Ti specimens contaminated with salivary pellicle and subsequently treated with commonly used decontamination solutions. Panel (A) depicts the total bacterial load per surface unit, whereas Figure 1B shows the bacteria live/dead ratio. Letters in the bar chart indicate significant differences from control (a), saliva (b), chlorhexidine (c), citric acid (d), essential oil‐based mouthwash (e), PBS (f), and phosphoric acid (g), *p* < 0.05. (C) Fluorescence microscope photographs of titanium specimens representative of the different groups.

Among all six aqueous solutions tested, CA, essential oil‐based mouthwash, PBS, and PA showed some effectiveness in reducing the bacterial load on the titanium surface. However, none of them were able to completely restore the surface to the pre‐contamination levels (Figure 5A). Furthermore, CHX, CA, and essential oil‐based mouthwash demonstrated better bactericidal ability, whereas saline showed low antimicrobial activity (Figure 5B,C).

XPS reveals that all six solutions were able to substantially reduce the surface content carbon on the saliva‐contaminated samples, with CA and saline being the most effective (Figure 4B). CA and saline were the only solutions that markedly reduced surface nitrogen (Figure 4C). However, none of the solutions could fully restore the surface titanium and oxygen elements back to the uncontaminated levels (Figure 4D,E). The BCA test indicated that CA (*p *= 0.012) and PA (*p* = 0.016) were able to significantly reduce the protein contamination from the surface (Figure 2).

These findings indicate that among the commonly used tested solutions, CA is the most effective in decontaminating the titanium surface. However, it is important to note that none of the six test solutions were able to completely eliminate the salivary pellicle or microbial compounds.

### 3.3 Effect of Detergents and Solvents on the Salivary Pellicle

**Figure 6:**
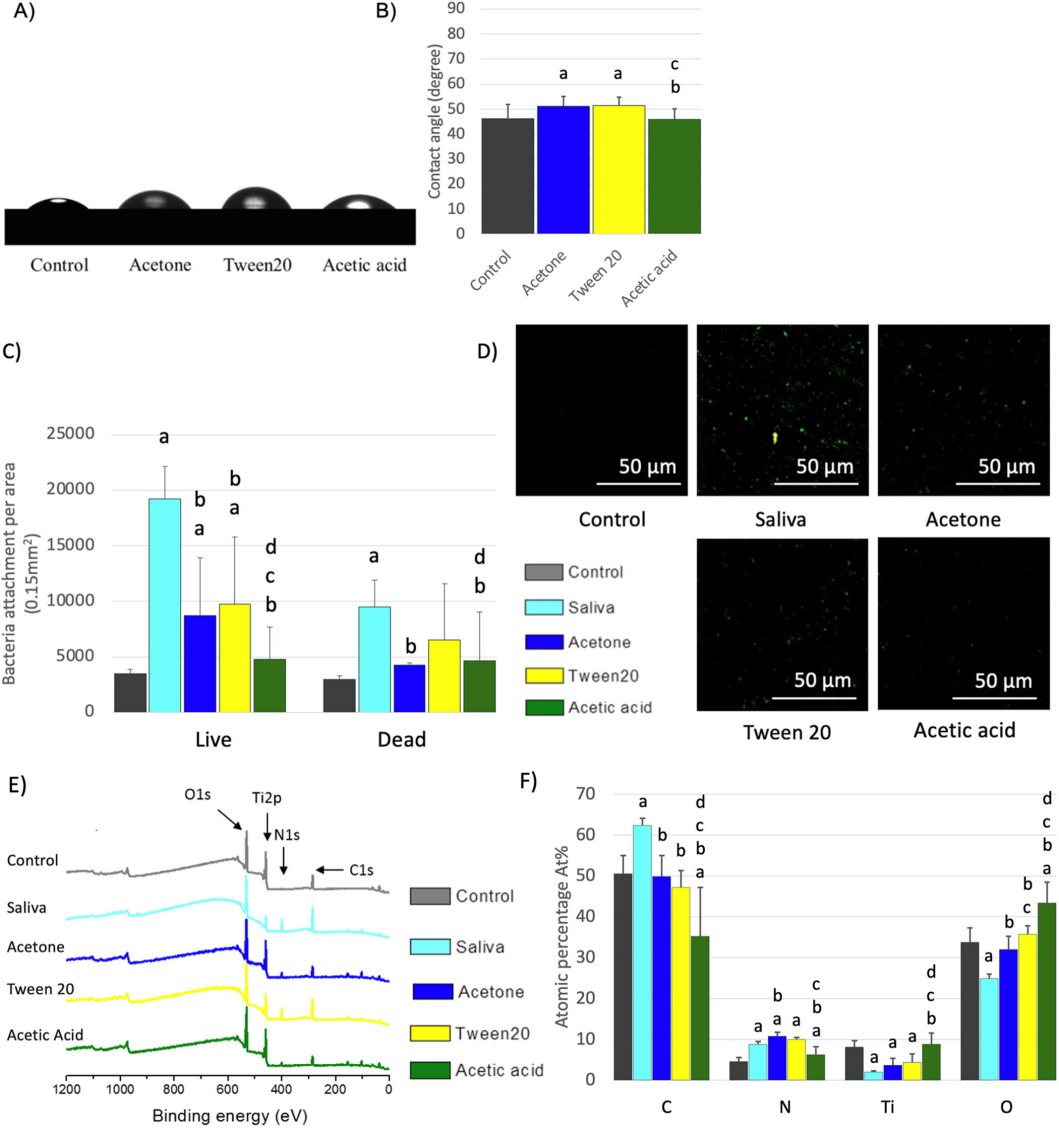
Effect of hydrophobic and amphiphilic chemical treatments on the surface composition and bacterial load of titanium contaminated with salivary pellicle. (A) Photographs of a contact angle experiment showing water droplets placed on clean Ti (control), pellicle‐contaminated Ti, and pellicle‐contaminated titanium treated with a solvent (Acetone) and a surfactant (Tween 20). (B) Bar charts depicting the measurements of the contact angle experiment; letters indicate significant differences in contact angle from control (a), acetone‐ (b), Tween 20‐ (c), and acetic acid‐treated surfaces, respectively (*p* < 0.05). (C, D) Fluorescence microscopy analysis of the microbial load on pellicle‐contaminated Ti‐treated with solvents and a surfactant. Letters in the bar chart indicate significant differences from control (a), saliva (b), acetone (c), Tween 20 (d), and acetic acid (*p* < 0.05). (E) XPS survey spectra of the different surfaces: control, saliva, Tween 20, and acetic acid. Peaks of the elements carbon (C1s), nitrogen (N1s), oxygen (O1s), and titanium (Ti2p) are shown. (F) Bar chart with letters showing significant differences of the surface elemental composition from control (a), contaminant (b), acetone (c), Tween 20 (d), and acetic acid groups, respectively (*p* < 0.05).

The decontamination solutions tested above were unable to completely remove surface contaminants. The above experiments showed that the salivary pellicle rendered the titanium surface more hydrophobic, which would explain the limitation with the above solution. Based on these observations, we hypothesized that organic agents capable of dissolving lipophilic compounds might be effective in salivary pellicle removal. To test this hypothesis, we tried three different agents: a very potent organic solvent (acetone), a food‐grade solvent (acetic acid), and a medical‐grade detergent (Tween 20). All three agents successfully increased the surface wettability on the saliva‐coated titanium, with acetic acid being the most effective (Figure 6A,B). Fluorescence microscopy demonstrated that the tested solvents and detergent reduced the overall bacterial load on saliva‐coated titanium, with acetic acid being the only treatment that reduced both live and dead bacteria back to the baseline control levels (Figure 6C,D). XPS analysis revealed that all three treatments were effective in significantly reducing the concentration of carbon and increased the oxygen on the surface contaminated with the saliva. However, only acetic acid was able to recover surface titanium levels and reduce surface nitrogen contamination compared to the saliva group (Figure 6E,F). These findings align with the results of the BCA test, where acetic acid was the most effective among the three agents in removing surface proteins. However, none of them could eliminate the proteins from the titanium surface contaminated with saliva (Figure 2).

### 3.4 Biofilm‐Coated Titanium Decontamination

Based on the initial findings of removal of the salivary pellicle, we decided to test the effectiveness of citric acid (an aqueous solution) and acetic acid (an organic solvent), and their combination in removing pathological biofilms from titanium surfaces.

**Figure 7:**
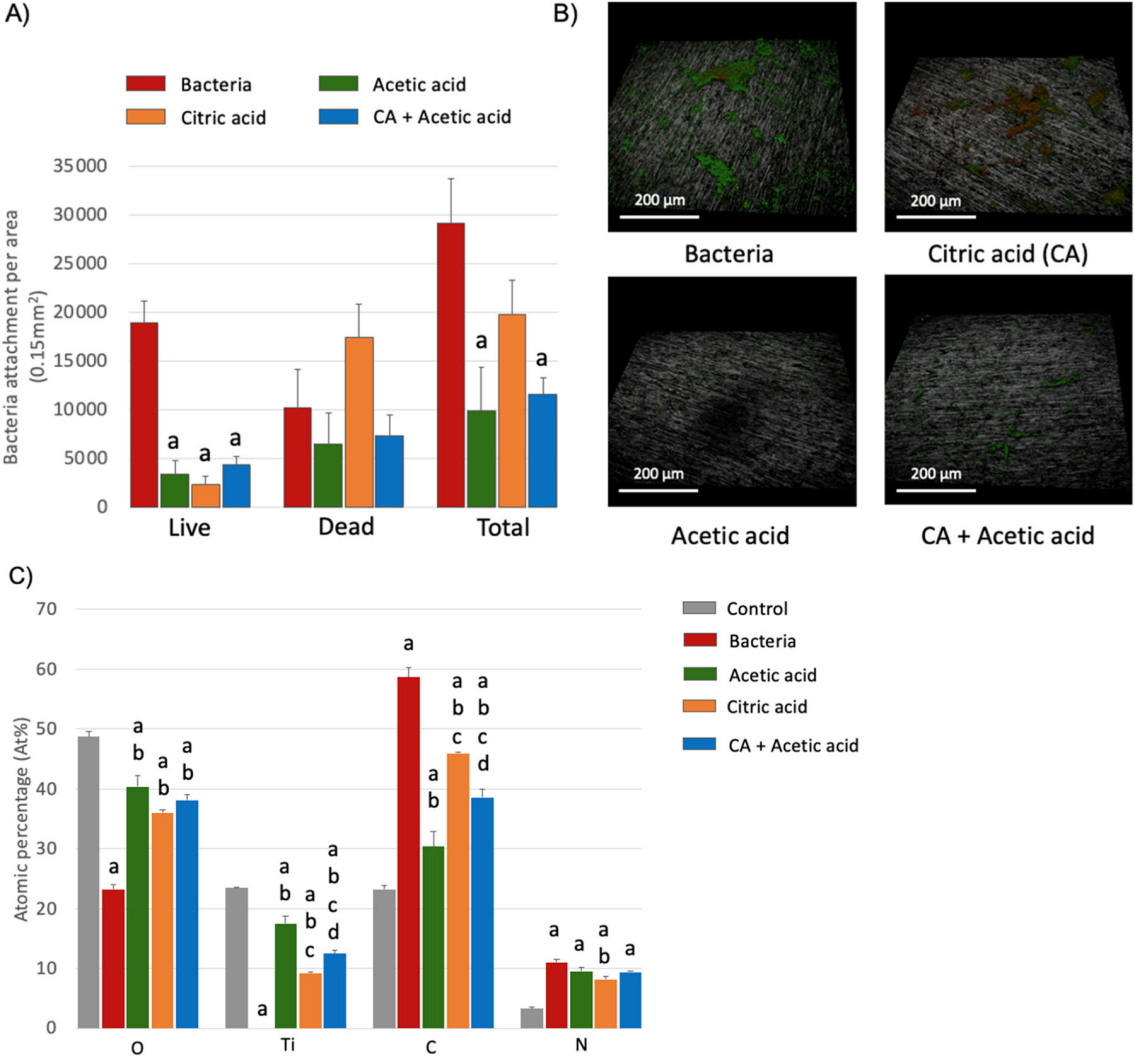
(A/B) Fluorescence microscopy analysis to assess the bacterial load on the Ti specimens. Letters in the bar chart indicate significant differences from control (a), bacterial biofilm (b), acetic Acid (c), citric acid (d), and citric acid + acetic Acid (e), *p* < 0.05. (C) XPS survey spectra of the different surfaces: polished pure Ti disc, bacterial‐contaminated Ti, and its treatment with cleaning agents: acetic acid (15 min), citric acid (15 min), or citric acid (7.5 min) and then acetic acid (7.5 min).

Fluorescence microscopy revealed that both CA and acetic acid were significantly effective in reducing the levels of live bacteria, although CA displayed the strongest bactericidal effect (*p* = 0.006). Samples treated with acetic acid alone or in combination with CA showed a notable reduction in the surface bacterial load on the biofilm‐coated titanium surfaces (Figure 7A,B). All three of the treatment groups performed well in reducing surface carbon and nitrogen, along with the recovery of surface oxygen and titanium. The acetic acid treatment displayed the best performance in removing carbon and restoring titanium and oxygen levels, whereas the use of citric acid alone was the most effective in removing surface nitrogen (Figure 7C). Nevertheless, the combined use of citric acid with acetic acid did not improve the performance compared to the use of these treatments separately.

## 4 Discussion

The salivary pellicle forms rapidly on a titanium surface exposed to saliva (C. Hannig et al. 2007). Once formed, the pellicle remains strongly attached to the titanium surface even after ultrasonication in distilled water. This indicates the tenacity of the pellicle and the challenge in decontaminating titanium implants. Indeed, our findings demonstrate that many chemical solutions commonly used for managing peri‐implantitis cannot fully remove the salivary pellicle and the bacteria adhered on titanium surfaces. This could be attributed to the hydrophobic nature of the titanium saliva‐coated pellicle, which renders it resistant to water‐based hydrophilic treatments. In contrast, detergents and solvents such as acetic acid are more effective in cleaning these water‐repellent contaminants on saliva‐coated titanium surfaces. In our experiments on titanium contaminated with pathological biofilm, acetic acid alone or in combination with citric acid demonstrated good performance in eliminating titanium biofilm.

### 4.1 Hydrophobicity of Saliva‐Coated Titanium

The increased contact angle observed for saliva‐coated titanium indicates enhanced hydrophobicity, likely due to the accumulation of lipophilic organic compounds such as carbon (C─C). The XPS data suggest that the salivary pellicle possibly comprises proteins, lipids, or carbohydrates such as glycoproteins and glycolipids (Lendenmann, Grogan, and Oppenheim 2000). The presence of glycoproteins and glycolipids is supported by peaks in the C1s spectrum that correspond to carbon single‐bonded to oxygen or nitrogen and ester bonds. These observations align with previous studies that have shown how the pellicle can enhance the hydrophobicity of dental materials like PMMA (polymethyl methacrylate) (Sipahi, Anil, and Bayramli 2001). This increased hydrophobicity might be attributed to the lipids, carbohydrates from saliva, or polysaccharides from bacteria, which contribute to the water repellency of the pellicle (Epstein et al. 2011; Matczuk et al. 2017).

### 4.2 Aqueous Solutions for Salivary Pellicle Removal

Given the hydrophobic nature of the pellicle, it would be expected that water‐based solutions alone may not eliminate completely it from titanium surfaces. Therefore, the development of novel decontamination procedures for titanium should consider the hydrophobic nature of surface contaminants. Among the aqueous titanium tested on saliva‐coated titanium, it was found that CHX, CA, and essential oil‐based mouthwash demonstrated superior bactericidal capacity; CA, essential oil‐based mouthwash, PBS, and PA were most effective in reducing the surface bacterial load; and CA and saline significantly removed carbon and nitrogen contaminants, whereas CA and PA effectively eliminated surface proteins. CA performed exceptionally well among all tested aqueous titanium. However, none of them could completely remove salivary contaminants and bacteria. The bactericidal effect of CHX and essential oil‐based mouthwash was expected based on their well‐known antimicrobial properties (Mombelli 2002; Kato et al. 1990). CHX and essential oils are primarily utilized as adjunctive therapies alongside routine professional mechanical submucosal debridement and mechanical oral hygiene practices. Their antimicrobial properties aid in reducing the bacterial load of the microbial biofilm, thereby mitigating gingival inflammation and supporting overall periodontal health. This highlights their role as a supportive measure rather than standalone treatment (Quintas et al. 2015). On the other hand, the antimicrobial and protein removal capabilities of CA and PA could be attributed to their ability to chelate calcium ions (Sousa and Silva 2005; Chockattu, Deepak, and Goud 2017; Pérez‐Heredia et al. 2008). The presence of Ca^2+^ facilitates the adsorption of salivary components to titanium by acting as a protein crosslinker (Klinger et al. 1997; Steinberg et al. 1995). Conversely, Ca^2+^ chelation by CA and PA undermines the mechanical properties of the salivary pellicle and breaks down its structure (Proctor et al. 2005). The concentration of CA, however, plays a critical role in balancing its antimicrobial effects, surface decontamination efficacy, and potential adverse effects on titanium surfaces. Investigating the use of CA (40%) for removal of in vitro and in vivo oral biofilm formed on titanium surface, it was found that it significantly reduced biofilm (~5‐log reduction) in situ compared to the control group, with no difference between application methods (immersion vs. rubbing) (Souza et al. 2019). Higher CA concentrations (40%) resulted in significant surface alterations, including increased titanium surface roughness and wettability, enhancing biofilm removal (Cordeiro et al. 2021). However, lower concentrations of CA (10%) showed effective antibacterial action and biofilm removal when applied by rubbing for 4 min, with no observed cytotoxic effects. Conversely, 20% and 40% CA showed moderate‐to‐severe cytotoxicity, highlighting the importance of balancing efficacy with biocompatibility. The pH of lower CA concentrations (> 6.3) was considered nontoxic, maintaining fibroblast attachment and viability (Cordeiro et al. 2021). The findings from our study complement the existing literature by confirming the robust efficacy of 50% CA in biofilm removal while providing insights into its potential for restoring surface composition. Nevertheless, as higher CA concentrations may alter the titanium surface, future research comparing CA at varying concentrations will be critical to identify an optimal concentration that balances biofilm removal, surface preservation, and biocompatibility.

However, the incomplete removal of bacteria is likely due to the different mechanisms involved in salivary adherence and microbial attachment. The limited effectiveness of these treatments in removing the salivary pellicle and initial microbial adhesion on titanium surfaces raises concerns about their applicability in managing more complex biofilms in titanium implants. For example, although chlorhexidine effectively killed surface bacteria, it failed to eliminate the pellicle components from the surface.

### 4.3 Effect of Organic Solvents and Detergents on Salivary Pellicle Removal

Given the hydrophobic nature of the salivary pellicle forming on titanium surfaces, we investigated the effectiveness of solvents and a detergent in removing the pellicle and microbial components. Our findings supported the hypothesis that organic agents capable of dissolving hydrophobic compounds can effectively decontaminate the titanium surface. In particular, acetic acid significantly reduced the surface bacterial load (both live and dead) and restored the surface elemental compositions levels back to the uncontaminated baseline levels, reducing the hydrophobic C–C contaminants, thus leading to the recovery of surface wettability. Acetic acid is a weak carboxylic acid with antibacterial and antifungal properties that has a long history of sporadic use in medicine over the past 6000 years (Ryssel et al. 2009). Its efficacy against bacteria can be attributed to its acidity and its ability to cross the bacterial membranes due to the equilibrium between its ionized and non‐ionized forms (Hirshfield, Terzulli, and O'Byrne 2003; Warnecke and Gill 2005). The non‐ionized form freely diffuses through hydrophobic bacterial membranes (Walter and Gutknecht 1984; Slonczewski et al. 2009), contributing to acid‐induced protein unfolding and membrane damage when internalized into the cytoplasm (Lund, Tramonti, and De Biase 2014; Halstead et al. 2015).

Tween 20, a non‐ionic surfactant used in domestic and pharmacological applications (Hadi et al. 2010), is a biocompatible agent known for solubilizing peripheral proteins from cell membranes (Pou De Crescenzo et al. 2001). It is frequently utilized in the formulation of biotherapeutic products to prevent protein surface adsorption and aggregation (Kerwin 2008). Although not as effective as acetic acid in reducing the surface hydrophobicity and the microbial load, Tween 20 still achieved significant decontamination by recovering surface carbon and oxygen to the baseline pre‐contamination levels. Acetone, as a standard hydrophobic solvent, effectively removed the saliva contaminants from the titanium surface, further supporting our hypothesis on the hydrophobic properties of the pellicle.

It is noteworthy that the detergent and solvents tested demonstrated limited effect in removing surface proteins. This implies that they mostly interact with the hydrophobic components of the pellicle, such as lipids, bacteria, or lipopolysaccharides, rather than directly affecting the salivary proteins on the titanium surfaces.

### 4.4 Removal of Pathological Biofilm

In the experiment discussed above, acetic acid and citric acid were identified as promising treatments for titanium decontamination. Accordingly, these solutions were tested on titanium contaminates with a pathological biofilm. The biofilm model used for this purpose consisted of six oral bacteria species (standard reference strains), and the model has been validated in numerous investigations focused on biofilm removal from teeth and implant surfaces (Sánchez et al. 2014; Fernández et al. 2017). The application of both citric acid and acetic acid to remove the 72 h biofilm from titanium surfaces led to substantial restoration of the surface titanium oxide layer. This implies that both solutions have the potential to effectively eliminate surface biofilm, although acetic acid demonstrated superior biofilm removal capabilities. It not only restored the titanium surface elements closer to its uncontaminated state but also significantly reduced the surface bacterial load. These findings indicate that acetic acid holds promise as an antibiofilm agent. This is in agreement with a previous study reporting the ability of acetic acid to remove mature skin biofilms and promote wound healing (Bjarnsholt et al. 2015).

On the other hand, citric acid displayed better bactericidal effect than acetic acid. This can be attributed to the strong antimicrobial properties of citric acid (Su et al. 2014; Ogita, Fujita, and Tanaka 2009). Additionally, citric acid significantly reduced surface nitrogen due to its Ca^2+^ chelator effect, which hinders protein attachment.

In summary, the study results indicate that both citric acid and acetic acid have potential applications in biofilm removal from titanium surfaces. Acetic acid showed superior biofilm removal and restoration of the surface oxide layer, whereas citric acid showed better bactericidal effects and the ability to impede protein attachment.

### 4.5 Clinical Implications of Our Findings

Our study carries significant clinical implications. First, we demonstrated that six solutions commonly used for the management peri‐implantitis were unable to completely remove surface contamination caused by exposure to saliva. This finding indicates that none of those solutions alone is able to achieve the predictable outcomes in peri‐implantitis treatment (Carcuac et al. 2015). Second, our findings present an opportunity for the development of novel chemical treatments based on a better understanding of the hydrophobic nature of titanium when contaminated by saliva and pathological biofilms. By using organic solvents that are more effective in removing the hydrophobic components of the pellicle and the bacterial biofilm, we can overcome the limitations of currently used chemical solutions. Tween 20 and acetic acid have already been recommended for other biomedical cleaning procedures. For instance, acetic acid solutions have been approved by the US Food and Drug Administration for bladder irrigation and treatment of external otitis (Bjarnsholt et al. 2015). However, our findings suggest for the first time that these agents may also hold potential in preventing or treating peri‐implantitis. Further research would be necessary to explore this possibility in greater depth.

### 4.6 Limitations and Future Directions

The main limitation of this study was the fact that the experiments presented were all performed in vitro; thus, further clinical research is required to determine whether our observations would be extrapolated to clinical practice.

Another limitation of this study was that experiments were only performed on machined titanium. Surface characteristics of titanium implants and abutments can vary depending on their fabrication process. Machined titanium surfaces have regular lines or shallow pits resulting from the cutting process, whereas acid‐etched titanium implants have a more complex topography (Martinez‐Hernandez 2020). Therefore, our finding would have to be confirmed in treated surfaces in future studies. However, even though surface topography can influence salivary protein interactions on biomaterials (Galli et al. 2002), recent research suggests that the topographic differences between titanium surfaces may not be the determinant contributing to the specific formation of a salivary pellicle (Zuanazzi, Xiao, and Siqueira 2020).

Moreover, fluorescence microscopy provided a reliable method for visualizing and semi‐quantifying bacterial load, but additional techniques such as quantitative PCR (qPCR) could offer more precise quantification of microbial DNA and insights into specific bacterial species present. Future studies should incorporate qPCR to complement fluorescence microscopy and provide a more comprehensive evaluation of bacterial load and biofilm composition.

## 5 Conclusion

The salivary pellicle forms a hydrophobic coating of bacteria and organic molecules on the titanium surfaces that are resistant to aqueous decontamination treatments. Organic solvents and surfactants demonstrate superior performance in decontaminating the saliva coating compared to aqueous solutions. Among these agents, acetic acid showed prominent effects in eliminating bacterial load and restoring surface elemental composition to the uncontaminated level. The effect of acetic acid was further confirmed on titanium contaminated with multispecies pathological biofilms.

## Author Contributions

**Wenji Cai:** methodology, writing–original draft, investigation, and formal analysis. **Azam Fayezi Sisi:** investigation and formal analysis. **Mohamed‐Nur Abdallah:** investigation and formal analysis. **Ashwaq A. Al‐Hashedi:** investigation and formal analysis. **Juan Daniel Gamonal Sánchez:** formal analysis. **Enrique Bravo:** investigation. **Hasna H. Kunhipurayil:** writing–review and editing and formal analysis. **Rubens Albuquerque:** conceptualization, resources, and funding acquisition. **Zahi Badran:** writing–original draft. **Mariano Sanz:** conceptualization and supervision, **Faleh Tamimi:** conceptualization, writing–review and editing, and supervision.

## Conflicts of Interest

The authors declare no conflicts of interest.

## Supporting information

Supporting information.

## Data Availability

Data are available on request from the authors.
