# Supplementary material for: In Vitro Assessment of Salivary Pellicle Disruption and Biofilm Removal on Titanium: Exploring the Role of Surface Hydrophobicity in Chemical Disinfection
Source: Clin Exp Dent Res. 2025 May 8;11(3):e70082. doi: 10.1002/cre2.70082 (PMC12061848; doi:10.1002/cre2.70082)
Supplement: Supplementary file 1 — Supporting information. [file CRE2-11-e70082-s001.docx]

**Supplementary**


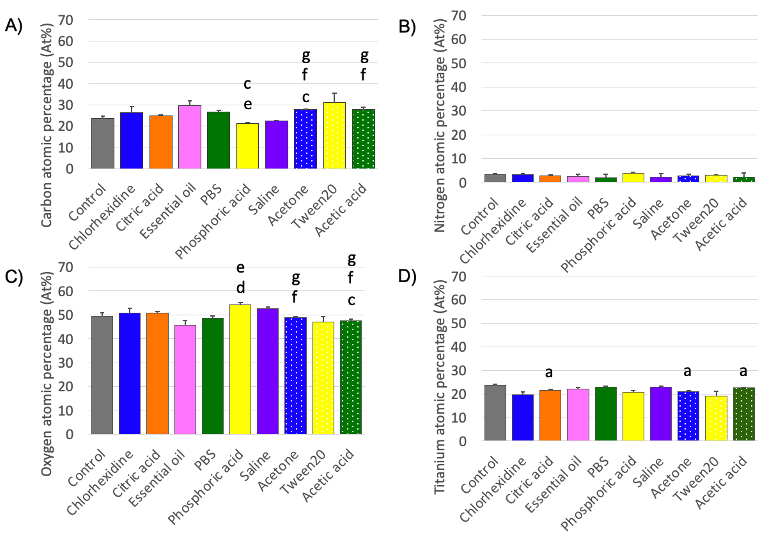


**Supplementary Figure 1:** Effect of chemical treatment on the surface of composition of clean uncontaminated titanium: A-D)atomic percentage of the elements on the clean uncontaminated Ti discs (control), exposure to different testing solutions; letters in the bard chart indicate significant difference from: control (a), chlorhexidine (b), citric acid (c), essential oil-based mouthwash (d), PBS (e), phosphoric acid (f), saline (g), acetone(h), Tween 20 (i), acetic acid (p less than 0.05)
